# A Supervised Approach to Quantifying Sentence Similarity: With Application to Evidence Based Medicine

**DOI:** 10.1371/journal.pone.0129392

**Published:** 2015-06-03

**Authors:** Hamed Hassanzadeh, Tudor Groza, Anthony Nguyen, Jane Hunter

**Affiliations:** 1 School of ITEE, The University of Queensland, Brisbane, QLD, Australia; 2 Garvan Institute of Medical Research, Darlinghurst, NSW, Australia; 3 The Australian e-Health Research Centre, CSIRO, Brisbane, QLD, Australia; University of Illinois-Chicago, UNITED STATES

## Abstract

Following the Evidence Based Medicine (EBM) practice, practitioners make use of the existing evidence to make therapeutic decisions. This evidence, in the form of scientific statements, is usually found in scholarly publications such as randomised control trials and systematic reviews. However, finding such information in the overwhelming amount of published material is particularly challenging. Approaches have been proposed to automatically extract scientific artefacts in EBM using standardised schemas. Our work takes this stream a step forward and looks into consolidating extracted artefacts—i.e., quantifying their degree of similarity based on the assumption that they carry the same rhetorical role. By semantically connecting key statements in the literature of EBM, practitioners are not only able to find available evidence more easily, but also can track the effects of different treatments/outcomes in a number of related studies. We devise a regression model based on a varied set of features and evaluate it both on a general English corpus (the SICK corpus), as well as on an EBM corpus (the NICTA-PIBOSO corpus). Experimental results show that our approach performs on par with the state of the art on the general English and achieves encouraging results on the biomedical text when compared against human judgement.

## Introduction

Evidence Based Medicine (EBM) is a prescribing scenario that employs available medical research outcomes in the treatment process. One of the governing methods used by EBM to summarise the state of the art in a particular area is to compile systematic reviews. As shown in [1], this process starts with searching for studies relevant to a predefined review question, followed by a selection process that retains only the publications meeting the criteria for inclusion in the review. Selecting the most relevant publications, among often thousands from the search results, is a time consuming task for experts. Moreover, the key statements externalized by the resulting set (e.g. Outcome or Intervention) need to be collated in order to provide a comprehensive, succinct and balanced overview of the domain.

The complexity and effort associated with this process leads to the need of devising automated methods that aid, in particular, the retrieval and consolidation of the relevant key statements. For example, consider the two following outcome statements:

- **Outcome A:** No clinically relevant adverse events, such as urinary retention, respiratory disturbances, or wound infections were reported in the M-ADL group.
- **Outcome B:** Neither intra-operative nor post-operative clinically relevant adverse events, such as urinary retention, respiratory disturbances, or wound infections, were observed.

Starting from the case context (i.e., *urinary retention, respiratory disturbances* and *wound infections*), using only the existing search engines, it is very difficult to find the most relevant related statements and publications. It is a challenging task, firstly because of the sheer volume of existing literature and secondly because of its intrinsic complexity—rooted in defining computationally the appropriate semantic similarity between such statements.

A framework for measuring the semantic similarity of sentences from scholarly publications can leverage the process of finding related evidence. By obtaining the conceptual relatedness of the sentences that carry a key rhetorical role in the EBM domain, clinicians can easily track the evolution of a particular intervention (for instance one of the previously mentioned examples) in a number of relevant studies. Furthermore, this lays the foundation for building semantically consolidated networks of scientific artefacts, and inherently scientific publications.

Scientific artefacts—the essential knowledge emerging from the described research—vary in terms of granularity and level of abstraction. From the granularity perspective, they may cover an entire sentence, or a part of it—e.g., a phrase or a clause [2]. Similarly, the level of abstraction ranges from high-level, generic types, such as Method and Results [3] to fine-grained decompositions, like Hypothesis, Discussion, Observation [4], or to domain-specific ones—e.g., Intervention, Population, Outcome [5]. Recognising and classifying these scientific artefacts is challenging, however, the literature consists of several approaches that achieve reasonable results on automatically identifying them. Moreover, in the EBM domain, classification results are particularly encouraging, as shown in [2, 6–8]. Using the extraction methods and results as foundation, we can now focus on investigating semantic relationships between artefacts, as well as appropriate consolidation approaches. The contribution of this paper is two-fold: firstly, we propose an efficient regression model for computing the pairwise similarity between statements, relying on the assumption that the statements represent full sentences. Additionally, as part of the model, we include a series of similarity features from new perspectives, such as, window-based syntactic and semantic similarity measures and sentence structural similarities. Secondly, we make the first steps towards understanding the difference between applying such a model trained on a general English corpus to a specific EBM corpus.

The experimental results achieved on the SICK corpus [9] are comparable to those achieved by the state-of-the-art solutions. On the other hand, given the lack of an appropriate gold standard in the EBM domain, we have validated the application of our approach on a subset of the NICTA-PIBOSO corpus [6] with the help of five human annotators. The outcome shows that, even though the model has been built using general English features, the performance is still encouraging when correlated with human judgment.

## Materials and methods

The task we aim to address is to quantify the degree of similarity between pairs of sentence-level scientific artefacts sharing the underlying rhetorical type—e.g., Population, Intervention, Background, Study Design, or Outcome in the case of the PIBOSO scheme [6]. There are several ways to attain this goal, ranging from the ratio of directly aggregated (e.g., averaged) values of a set of features to compositional distributional semantics. We chose to approach this problem from a classification perspective. More concretely, given a pair of sentences, we encode a variety of relatedness features in a vector of attributes and then predict the degree of their similarity by employing learning algorithms.

In this setting, the complexity of the task is dictated by the granularity of the target classes. The most straightforward option would be to treat similarity as a dichotomy and classify the pair as being ‘similar’ or ‘not similar’. Increasing the level of granularity—i.e., instead of two classes to opt for three or more, e.g., ‘not similar’, ‘slightly similar’ and ‘similar’—leads to an increased difficulty in classification, since the boundaries between classes become more volatile. Finally, the choice of classes is also influenced by the availability of a gold standard and its associated annotation scheme. As we discuss in the Experimental Setup section, we were guided by the scheme used to annotate the SICK corpus [9]. This quantifies similarity as a real number ranging from 1 to 5, with 1 being completely dissimilar and 5 representing perfect similarity.

The features used for classification can be grouped into three major categories: Syntactic, Structural and Semantic Similarities. Numerical values of these features form a vector, which is then used as the corresponding instance, for a pair of sentences, in training a regression model. Table 1 summarises all features used in this study, indicating their category association, as well as their use in existing sentence-based similarity solutions. Below we discuss and exemplify each feature. Furthermore, the Supporting Information S1 Text provides a glossary of technical terms used to describe them.

**Table 1:** Features used to encode pairwise sentence similarity as a basis for the learning model.

**Feature Sets**	**Features**	**Measures and their usage in existing solutions**
Syntactic Similarity	Naive	Bags of words overlap (1 feature)—[10–12]
		Bags of lemmatised/stemmed words overlap(2 features)
		Set similarity of lemmatised effective words (1 feature)
		Jaccard similarity of set of words/lemmas (2 features)—[10]
		Cosine similarity of vectors of lemmatised effective words (1 feature)
	Window-based	Windows of words overlap (1 feature)
		Size of the longest shared window of words (1 feature)
		Windows of effective words overlap (1 feature)
		Size of the longest shared window of effective words (1 feature)
		Windows of POS tags overlap and longest overlapped windows (2 features)
	Other	Ratio of shared skipped bigrams (1 feature)—[13]
		Pairwise sentence polarity (1 feature)—[10, 12]
		Ratio of sentence lengths (1 feature)—[10, 11, 13]
Structural Similarity	Sentence Structure	Ratio of number of clauses (1 feature)
		Reduced parse tree overlap (1 feature)
Semantic Similarity	Basic	Role-based word-by-word similarity (3 features)—[13, 14]
		Semantic similarity of effective words (1 feature)—[10]
		Cosine similarity Information Content (IC) vectors (1 feature)
		Role-based Part of Speech (POS) tags alignment (2 features)
	Synonymy	WordNet-based synonym similarity (1 feature)—[12]
		FrameNet-based synonym similarity (1 feature)
	Sense Disambiguation	Normalised set similarity of best senses (2 feature)—[11]
		Category level similarity of best senses (2 features)
		Normalised set similarity of the best sensesof skipped bigrams (1 feature)
	Vector Space Model	Similarity of Sets of Associated Terms (1 feature)
		Cosine Similarity of Matrices ofAssociated Terms Vectors (1 feature)—[10–13]

Citations denote existing systems that have employed the corresponding features.

### Syntactic similarity measures

#### Bags of words overlap

A simple measure for computing the similarity of a sentence pair is the number of words they have in common. Although a pair of sentences with the same bag of words may convey completely different concepts, this measure along with a structural similarity measure can form an effective criterion for semantic comparison. We calculate this naive word-by-word similarity (overlap) measure by dividing the number of equal words in both sentences by the length of the longest one in a pair, regardless of their locations. Furthermore, we use the original lexical and morphological forms of the words—as they appear in the sentence. If *S*_1_ and *S*_2_ are two sentences, we denote their naive word-level similarity by *ν*(*S*_1_, *S*_2_) and defined it as shown in Eq 1.

$$\begin{array}{c} \nu \left(S_{1},S_{2}\right)=\frac{\sum_{w_{i}\in S_{1}}\{\begin{array}{cc} 1 & \text{if}\;w_{i}\;\in \;S_{2}\\ 0 & \text{Otherwise} \end{array}}{Max(|S_{1}|,|S_{2}|)} \end{array}$$ (1)

In the above equation *w*_*i*_ denotes each word in the bag of words of *S*_1_, while ∣*S*_1_∣ and ∣*S*_2_∣ denote the size, in number of words, of *S*_1_ and *S*_2_, respectively. The two sample sentences introduced in the Introduction (i.e. Outcome A and Outcome B) share 14 terms in common (based on their bags of words), with the longest sentence being Outcome A (20 words). Hence, *ν*(*S*_1_, *S*_2_) = 0.7 for this pair.

#### Bags of lemmatised/stemmed words overlap

The value of this feature is computed using the same method as above, however, instead of using bags of words, it uses bags of lemmas / stems. Using the root forms produced by lemmatisation / stemming enables us to align words that would otherwise be omitted from the direct word-by-word comparison (e.g. “infection” and “infections” would be aligned as both words would have a common stem of “infect”). Using Eq 1, the value of this measure for the pair of sample sentences is 0.7—in this particular case, the same as the bags of words overlap.

#### Set similarity of lemmatised effective words

There are a number of words in a sentence that do not play a major role in understanding the core meaning of the sentence—such as, determiners (*the*, *a*, *an*) and sometimes prepositions or subordinating conjunctions (*in*, *on*). Removing these leads to creating a bag of effective words. This feature computes similarity based on the set (and not the bag) of lemmatised effective words. If *L*_1_ and *L*_2_ are the sets of the lemmatised effective words in *S*_1_ and *S*_2_ respectively, their similarity *l*(*S*_1_, *S*_2_) is calculated as defined in Eq 2.

$$\begin{array}{c} l\left(S_{1},S_{2}\right)=\frac{|L_{1}\cap L_{2}|}{Max(|L_{1}|,|L_{2}|)} \end{array}$$ (2)

where ∣*L*_1_∣ and ∣*L*_2_∣ denote the size of the two sets of lemmatised effective words. In our example, the sets of the lemmatised effective words are *S*_1_: {*event, retention, disturbance, wound, infection, be, report, group*} and *S*_2_: {*event, retention, disturbance, wound, infection, be*}. Hence, the value of this feature *l*(*S*_1_, *S*_2_) = 0.75.

#### Jaccard similarity of sets of words/lemmas

Using the same interpretations for sets and lemmas as above, this feature computes their Jaccard similarity—*J*(*S*_1_, *S*_2_)—as shown in Eq 3:

$$\begin{array}{c} J\left(S_{1},S_{2}\right)=\frac{|S_{1}^{set}\cap S_{2}^{set}|}{|S_{1}^{set}\cup S_{2}^{set}|} \end{array}$$ (3)

where $S_{1}^{set}$ and $S_{2}^{set}$ are the set of words of the two sentences. The same applies for computing the Jaccard similarity of the sets of lemmas, instead of words.

#### Cosine similarity of vectors of lemmatised effective words

Cosine similarity is a vector based measure that calculate the Euclidean distance of two vectors. In this feature, the strings of lemmas of effective words in pairs are transformed into vector space and then their Cosine similarity would be calculated following Eq 4.

$$\begin{array}{c} C\left(S_{1},S_{2}\right)=\frac{V_{1}.V_{2}}{\left|\right|V_{1}\left|||\right|V_{2}\left|\right|} \end{array}$$ (4)

where *V*_1_ and *V*_2_ are the vector of lemmas of the effective word of two sentences in a pair, and *V*_1_.*V*_2_ denotes the dot product of two vectors which is then divided by the product of their norms (i.e. ∣∣*V*_1_∣∣∣∣*V*_2_∣∣).

#### Windows of words overlap

Two sentences may be deemed related also based on the number blocks of words they share. Using a sliding window of different sizes—starting from a window of two words and increasing to the size of the smaller sentence in the pair, we compute the window-based bags of words overlap and retain the total number of equal windows (i.e., with the bags of words overlap equal to 1). Furthermore, as an additional feature we also retain the size of the longest window for which the pair of sentences was equal.

Since sentences vary in size, and in order to ensure an appropriate comparison, the window-based bags of words overlap and the size of the longest equal window measures are normalised. The former is normalised by the number of 2-combinations in a set of n (i.e., (n2)), where *n* is the size of the smallest sentence in the pair. The latter is normalised by the size of the smallest sentence. In the context of our example, normalised window overlap of words is 0.4348, while the normalised longest equal window size is 0.6667.

This feature may take several forms, based on the content of the windows. Two alternative options to considering words are: (i) taking into account effective words, and (ii) considering part of speech (POS) tags.

#### Ratio of shared skipped bigrams

Skipped bigrams are the pairs of words that are created by combining two words located in arbitrary positions in the sentence. The feature creates sets of such skipped bigrams as a basis for similarity comparison and therefore it encodes this similarity based on the number of equivalent pairwise bigrams. Skipped bigrams are formed from participating verbs, nouns, adjectives, and adverbs and compute the intersection of sets of these bigrams between the sentences. The final value of this measure is the size of the intersection set over the size of the longest set of skipped bigrams in a pair. For our example, this feature leads to a value of 0.6286.

#### Pairwise Sentence Polarity

This measure can be calculated by investigating the presence of some lexical elements that act as negation agent, e.g., *not, neither, no*, etc. We apply the NegEx algorithm [15] to find the negation in sentences and then perform pairwise comparison of the polarity of sentences. Our sample two sentences have the same polarity because they are negated with *No* and *Neither*. The value of this measure would be 0 if both sentences in a pair have the same polarity (i.e., positive-positive or negative-negative) and 1 otherwise.

#### Ratio of Sentence Lengths

This feature computes the ratio between the sizes of the two sentences based on their bags of words. The ratio divides the smaller size to the larger one. The value of this ratio for our two example sentences is 0.95.

### Structural similarity measures

Another dimension that enables us to decode the semantics of a sentence is its lexical structure. For instance, the meaning of a sentence can be inferred by aggregating the meaning of its composing clauses. Using this as a starting point, we have devised a series of measures that use the clause decomposition and their associated structures to encode the similarity between two sentences. It is worth mentioning that in the context of the Structural and Semantic similarity measures, the notions of “pre-verb component” and “post-verb component” refer to the left and the right components of the verb. The rationale behind this simplification is to provide crude triples that represent a sentence, which would then allow us to devise role-based and structural comparisons.

#### Ratio of number of clauses

**Fig 1:**
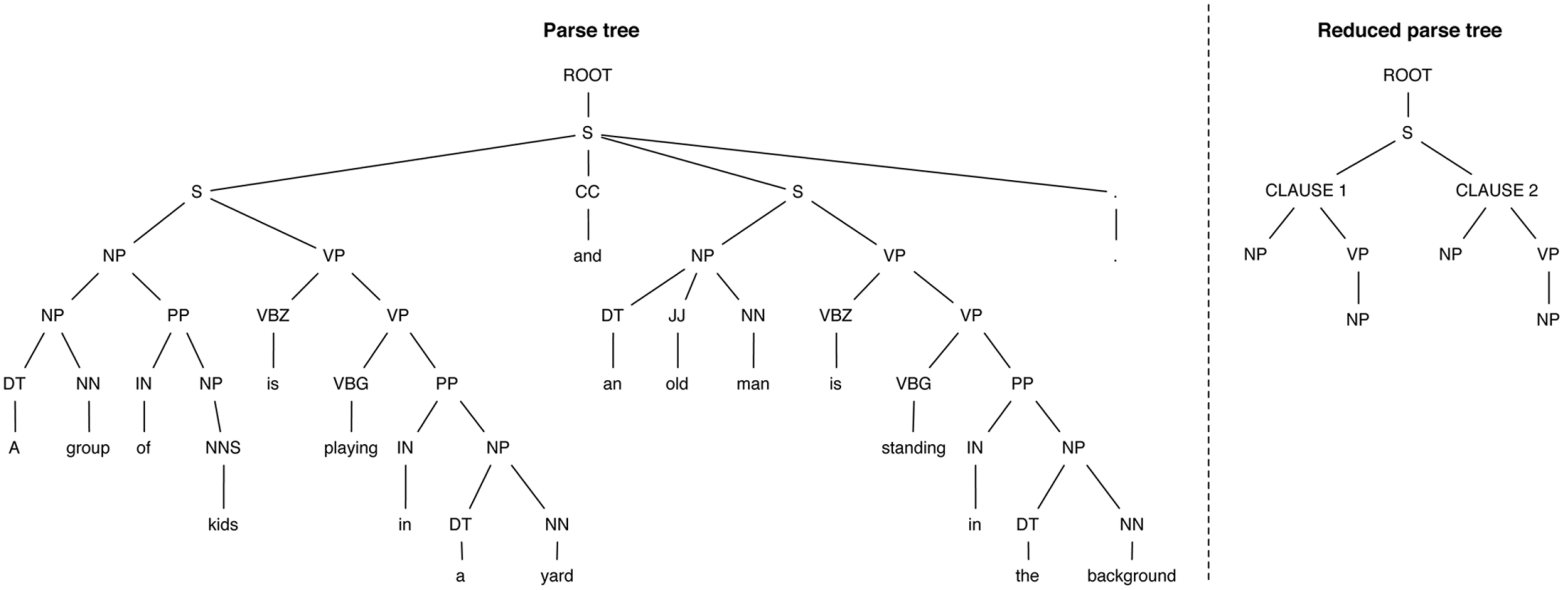
Example of parse tree and its reduced version for a sample sentence. The parse tree represents the syntactic structure of a sentence in the form of a rooted tree. The reduced form retains only the major groups of part of speech tags—i.e., NPs and VPs.

The parse tree of a sentence depicts the structure of the sentence and its constituent parts. The left side of Fig 1 exemplifies such a parse tree for the sentence “*A group of kids is playing in a yard and an old man is standing in the background*.” One of the main advantages when visualising this structure is that it provides quick access to the clauses composing the sentence—i.e., the elements denoted by *S* in the figure.

This measure is computed by analysing the parse tree of each sentence and finding the number of clauses they are composed of. The ratio divides the size in terms of clauses of the smaller sentence to the size of the longer sentence. If *C*_1_ and *C*_2_ is the set of clauses of *S*_1_ and *S*_2_, respectively, the clause-level equality is denoted by *κ*(*S*_1_, *S*_2_) and is calculated as in Eq 5:

$$\begin{array}{c} \kappa \left(S_{1},S_{2}\right)=\frac{Min(|C_{1}|,|C_{2}|)}{Max(|C_{1}|,|C_{2}|)} \end{array}$$ (5)

where ∣*C*_1_∣ and ∣*C*_2_∣ are the number of clauses of *S*_1_ and *S*_2_, respectively. Although the equality of the number of clauses does not provide an insight into the semantic similarity between the sentences, it does have a positive impact when joining it with appropriate semantic measures—introduced in the following section. The value of *κ*(*S*_1_, *S*_2_) for our sample pair of sentences is 1, as both sentences have the same number of clauses (i.e., one).

#### Reduced parse tree overlap

While the previous measure was considering strictly the shallow size-based comparison, this measure provides a more in-depth analysis of the structural similarity. More concretely, it quantifies the overlap of the parsed trees composed of only the Part of Speech (POS) tags of the effective words. The right side of Fig 1 depicts the reduced parse tree for the sentence shown on the left side. The goal of these reduced trees is to capture the scaffolding of the composing clauses, by taking into account only the major POS tags. It can be quickly observed how the full sentence is summarised by means of its noun and verb phrases.

The actual measure—denoted by *s*(*S*_1_, *S*_2_)—performs a clause-based comparison to find the number of identical sub-trees, i.e. *t*_*r*_ and $t_{r}^{\prime }$, as defined in Eq 6.

$$\begin{array}{c} s\left(S_{1},S_{2}\right)=\frac{\sum_{C_{i}\in S_{1}}\sum_{C_{j}^{\prime }\in S_{2}}(\frac{\sum_{t_{r}\in C_{i},t_{r}^{\prime }\in C_{j}^{\prime }}\{\begin{array}{cc} 1 & \text{if}\;t_{r}=t_{r}^{\prime }\\ 0 & \text{Otherwise} \end{array}}{|C_{i}\left|\times \right|C_{j}^{\prime }|})}{\left|C|\times \right|C^{\prime }|} \end{array}$$ (6)

**Fig 2:**
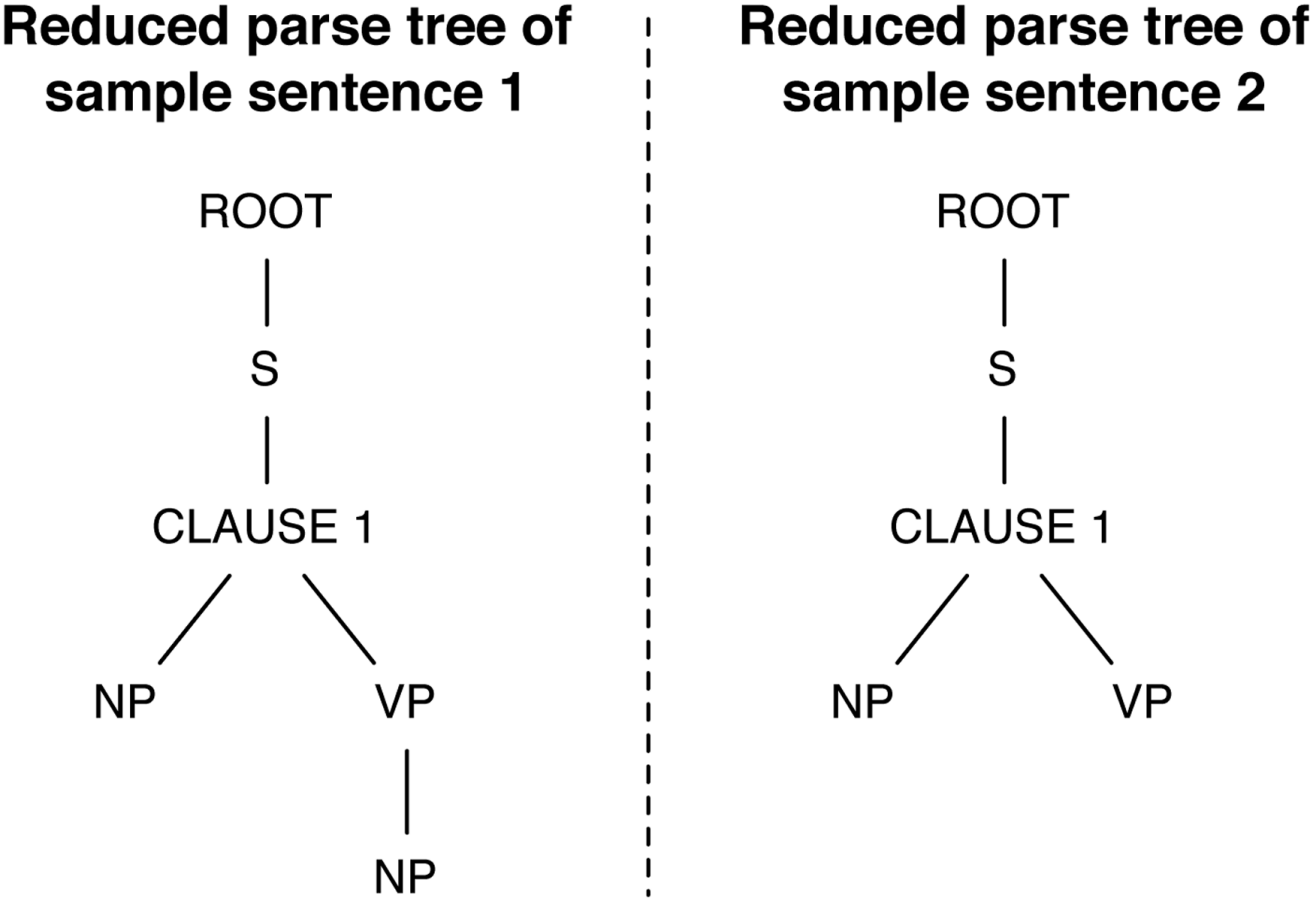
Reduced parse trees of the two sample sentences (i.e. Outcome A and B) listed in the Introduction.

In the above equation, ∣*C*_*i*_∣ and $|C_{j}^{\prime }|$ denote the number of words in the *C*_*i*_ and $C_{j}^{\prime }$ clauses respectively. Also, ∣*C*∣ and ∣*C*′∣ represent the number of clauses in *S*_1_ and *S*_2_. For our sample pair of sentences the value of *s*(*S*_1_, *S*_2_) is 0.67. The reduced parse trees of these two sentences are shown in Fig 2. It can be observed that the pre-verb components of the two sentences have identical structures, i.e., they are composed of one noun phrase. On the other hand, the pre-verb components are different since the first sentence provides more specific information via the post-verb component “*in the M-ADL group*”.

### Semantic similarity measures

The previous two sets of measures focus on morphological and structural characteristics of sentences. Although they play an important role in finding the relatedness of a pair of sentences, they fail to represent the underlying semantics of the sentences’ meaning. Here, we introduce a third set of measures that focus on semantics, with the help of some external knowledge resources.

#### Role-based word-by-word similarity

In order to compute this measure, we first split the sentences into clauses and determine the pre-verb component, predicate and post-verb component within each clause. Each of these roles is then transformed into a bag of lemmatised words, which is then compared to corresponding bags of lemmatised words denoting the same role in the other sentence. The similarity between the two bags of words is calculated using a mixture of two well-known semantic similarity measures—i.e., Lin [16] and Wu & Palmer [17], both having Wordnet [18] as background knowledge. The Lin similarity is defined as the ratio between the Information Content (IC) of the most informative common ancestor of two terms and the sum of the IC of the two terms. Wu & Palmer, on the other hand, define the similarity in terms of the path between the two terms in a given hierarchy, as well as the path between each of the terms and their Least Common Subsumer (LCS). In both cases, the IC and LCS are computed using Wordnet.

It is worth noting that the use of Wordnet for this role-based similarity computation results in appropriate values for pre-verb components and post-verb components composed largely of nouns and adjectives. Verbs, however, have a much lower coverage in Wordnet, which leads to the need for a different background knowledge when computing this similarity on predicates. Consequently, the predicate bags were compared using FrameNet [19] as background knowledge. Below we provide the actual formulation of the role-based word-by-word similarity measure.

If we denote the role-based similarity of two sentences by *r*(*R*_1_, *R*_2_), where *R*_1_ is the bags of words of a specific role in *S*_1_ (e.g., the bags of words of all pre-verb components in *S*_1_) and *R*_2_ is the bags of words of the same role in the *S*_2_, this measure is calculated as shown in Eq 7.

$$\begin{array}{c} r\left(R_{1},R_{2}\right)=\frac{\sum_{b\in R_{1}}\sum_{b^{\prime }\in R_{2}}Sim\left(b,b^{\prime }\right)}{|R_{1}\left|\times \right|R_{2}|} \end{array}$$ (7)

where *b* and *b*′ are bags of words carrying a particular role in *S*_1_ and *S*_2_ respectively, and *Sim*(*b*, *b*′) is different when comparing predicate bags and pre-verb component / post-verb component bags. For the latter, *Sim* represents the word-based average of the Lin and Wu & Palmer similarities—as per Eq 8.

$$\begin{array}{c} Sim\left(b,b^{\prime }\right)=\frac{\sum_{w\in b}\sum_{w^{\prime }\in b^{\prime }}(AVG(Lin\left(w,w^{\prime }\right)+WuPalmer\left(w,w^{\prime }\right)))}{\left|b|\times \right|b^{\prime }|} \end{array}$$ (8)

For predicate bags, *Sim* function uses FrameNet to find the relatedness of each pair of terms in two bags. In this case, if two terms share any common frame retrieved from FrameNet, then they are considered conceptually identical. This similarity measure can be formalised as in Eq 9.

$$\begin{array}{c} Sim\left(b,b^{\prime }\right)=\frac{\sum_{w\in b}\sum_{w^{\prime }\in b^{\prime }}\{\begin{array}{cc} 1 & \text{if}\;|w_{frames}\cap w_{frames}^{\prime }|>0\\ 0 & \text{Otherwise} \end{array}}{\left|b|\times \right|b^{\prime }|} \end{array}$$ (9)

where the *w*_*frames*_ is the set of related frames for term *w* in the FrameNet.

**Fig 3:**
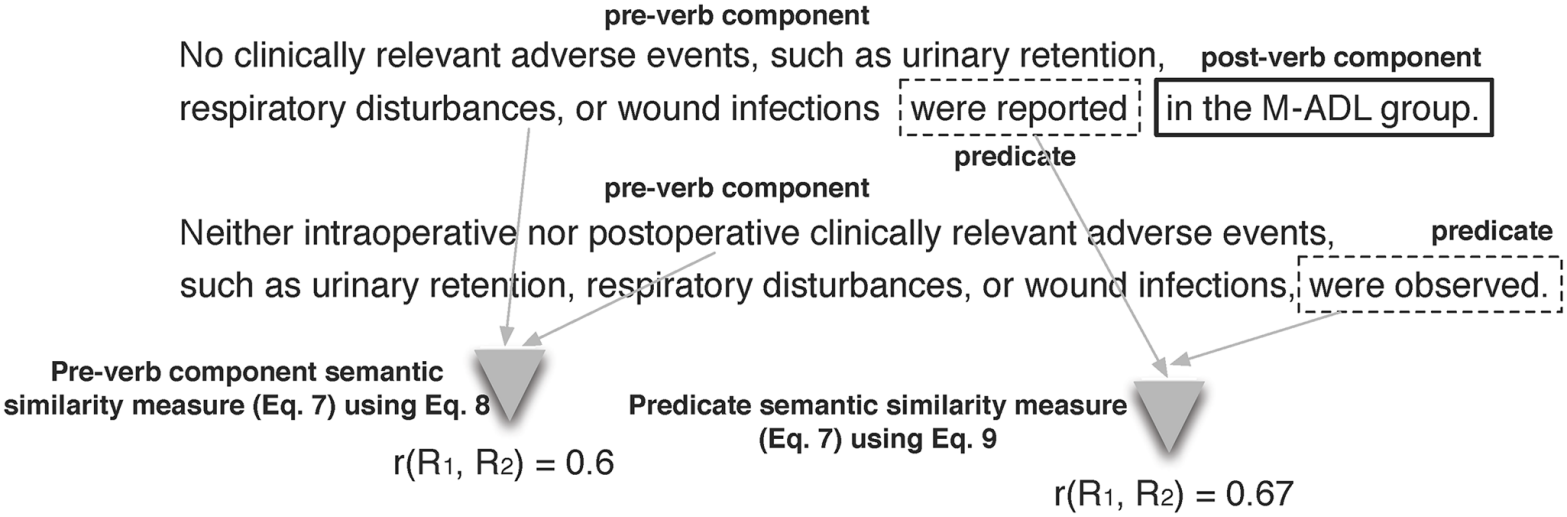
Example of role-based semantic similarity measure for two sample sentences. Both measures are computed using Eq 7, with the actual similarity being specific to pre-verb component (as defined in Eq 8) and predicates (as defined in Eq 9).

To give a concrete example, we illustrate the calculation of this measure for the two sample sentences depicted in Fig 3. It can be observed that each part of the sentences based on their pre-verb component / predicate / post-verb component are compared correspondingly. The second sentence does not have any post-verb component, hence the post-verb component similarity of this pair is 0.

#### Semantic similarity of effective words

Given the sets of effective words of a pair of sentences, we compute their similarity using the same method as above, however, without taking into account the underlying roles—i.e., it is computed in a sentence-wide manner. The actual semantic similarity measure uses, as in the previous case, different background knowledge for verbs and nouns.

#### Cosine similarity of IC vectors

Following a distributional semantics perspective, we map the sequence of words in a sentence to a vector of corresponding numeric values. In order to create this vector we use, again, the notion of Information Content (IC) [20]. The relatedness of a given pair of sentences can then be estimated by employing some form of distance measure between the two vectors, such as the cosine similarity. In our case, we employ pre-computed IC values using the SemCor corpus in WordNet Similarity Package [21]. Consequently, we lookup all lemmas associated with the words of a sentence and form the corresponding vector of IC values. The cosine similarity of the two sentence vectors provides the value for this measure.

#### Role-based POS tags alignment

POS tags associated with the effective words in the pre-verb components and post-verb components in a given sentence are concatenated to form a sequence of tags. The degree of similarity between the sequences is then computed by employing the Needleman-Wunsch algorithm [22]. The Needleman-Wunsch algorithm is an efficient approach for finding the best alignment between two sequences, and has been successfully applied in particular in bioinformatics to measure regions of similarity in DNA, RNA or protein sequences [23].

#### Wordnet-based synonym similarity

Synonyms provide a method to recognise shared meanings / semantic roots between several terms—e.g., one can identify a relationship between two terms by looking at the synonyms they share. This measure aims to quantify such a relationship by retrieving the Wordnet synsets associated with the lemmas of the effective words of a sentence and computing their normalised intersection. The normalisation is performed using the smaller of the two synsets.

#### FrameNet-based synonym similarity

Here, we apply the same principle as in the case of the previous measure by using FrameNet instead of Wordnet. We, hence, retrieve all the possible frames of each word in sentences and then compare the sets of frames of two sentences to find the number of shared frames. The normalised intersection is computed as above. Since we have used FrameNet to calculate the relatedness of the verbs of two sentences in the role-based word-by-word semantic similarity measure (described earlier in this section), here we only consider the terms belonging to the noun family.

#### Normalised set similarity of best senses

The meaning of the words is provided by the context in which they are placed; and vice-versa, the context defines a best sense / meaning for each of the words composing a sentence. This measure uses a Wordnet-based word sense disambiguation approach to find the best senses of the effective words. To give a concrete example, by using the Lesk algorithm [24] over the Wordnet dictionary, the best sense of the word “events” in the sample sentence “Outcome A” is the fourth sense, that is, “a phenomenon that follows and is caused by some previous phenomenon”, which can be encoded in form of “events#n#4” (i.e., it is in the form of a noun and the fourth sense better defines it in the context). These encoded best senses of the effective words are then used to create a set of senses for each sentence which can be applied as a measure of similarity of sentence pairs. The corresponding value is computed by performing set intersection of the best senses, normalised by the size of the smaller set.

In addition to the set of best senses for words of a sentence, we can retrieve all the relative synsets (i.e. a set of synonyms from Wordnet) of the best sense and then compute the set similarity of those synsets of a pair. The set of relative synsets is the list of synsets that are semantically related to the synset of a given word. For example, the synset of the fourth sense of the word “event” (as previously exemplified as the best sense for the sample sentence “Outcome A”) has 25 related synsets which are related to it by semantic pointers. The ID of the all related synsets of the best senses of words in a sentence are kept in a set which is then compared to the corresponding set of the another sentence to calculate the normalised set similarity.

#### Category level similarity of best senses

Synsets in Wordnet are organized into forty-five lexicographer files based on logical groupings, for example, animals, artefacts, feelings, events, person, etc. In this similarity measure, a set of these categories is created from the groups that the best senses of effective words in each sentence belong to. The normalised sets similarity for two sentences of a pair is then calculated by comparing the generated sets which are in category level (as opposed to the previous measure which is in synset level). In addition, in order to take into account the compositional characteristic of words in a sentence, we form a vector of the corresponding category names of effective words of sentences which their similarity can be computed using cosine similarity measure.

#### Normalised set similarity of the best senses of skipped bigrams

The syntactic comparison of skipped bigrams discussed earlier is expanded here at a more conceptual level. Instead of forming skipped bigrams using the words of a sentence, we create them from the semantic category of the best senses of words. As above, a word sense disambiguation approach is used to retrieve the best senses as well as the categories that they belong to and then to create the corresponding set of skipped bigrams. Subsequently, these sets of senses’ categories are used to compute the set intersection, normalised by the size of the smallest set.

#### Similarity of sets of associated terms

The literature consists of a series of well-established frameworks to explore a deeper understanding of the semantic relationship between entities, ranging from ontological reasoning to compositional as well as distributional semantics [25]. In the case of Distributional Semantics (DS), the meaning of a phrase is represented in a geometric space. Such a vector space model of meaning has been proposed under the assumption that, from a distributional perspective, words that occur in the same context tend to be semantically similar [26]. These distributional analyses of words are performed over very large corpora in which each word occurs in adequate numbers of contexts. The co-occurrence of each term and its associated terms are then captured and quantified in a numerical vector—usually with fixed dimension.

Our next two sets of features makes use of vector space models, built using Wikipedia English articles as the background corpus. The associated terms for each word in a sentence forms a set that can then be compared with a corresponding set of another sentence—for example, to calculate their intersection. The resulting value is normalised by the size of the largest set.

#### Cosine similarity of matrices of associated terms vectors

For this last feature, we use the numerical representation (vector) of each term, retrieved from the distributional model, to form a matrix of associated terms vectors for a sentence. To enhance the effectiveness of this similarity measure, only vectors of effective words of a sentence are used to build the matrix. Hence, the number of rows in this matrix denotes the number of effective words of the sentence, while the number of columns is the dimension of term vectors. Subsequently, we compute the cosine similarity of the matrices of a pair of sentences. The dimension of the term vectors is a fixed number which is set prior to the creation of the vectors. The column size of the matrices is hence the same, while the number of rows can be varied from sentence to sentence. To be able to calculate the dot product of the matrices, and consequently their cosine similarity, additional rows of zero values are augmented to the the smaller matrix whenever the dimension of a pair of matrices are not the same.

### Experimental setup

As mentioned in the previous section, the final similarity of a pair of sentences can be quantified in various ways—subject to the targeted degree of granularity. Given the lack of a gold standard in the biomedical (or EBM) domain, we chose to follow the approach proposed by the SemEval shared task 1 (ST1) [9], which focused on the evaluation of compositional distributional semantic models on full sentences. SemEval ST1 mapped the sentences similarity to a continuous value ranging from 1 to 5, with 1 representing the lower end and 5 the higher end of similarity. This scheme enabled us to treat sentence consolidation as a regression problem. In particular the values associated with the features described in the previous section (34 in total) have been aggregated into real-valued feature vectors and used to train different regression approaches, in addition to an ensemble of regressors.

Given the lack of an appropriate gold standard in EBM—or in the biomedical domain in general—we evaluated our approach using the SICK (Sentences Involving Compositional Knowledge) general English corpus [9]—also used in the SemEval 2014 ST1. SICK consists of 9,927 sentence pairs divided into two sets: training—5,000 pairs, and testing—4,927 pairs. Each pair of sentences was assigned a similarity score between 1 and 5. The corpus was created via crowdsourcing techniques, each pair of sentences being rated by ten annotators (using a 5-point Likert scale), with the final relatedness score representing the average of the ten values. The measure of (inverse) inter-annotator agreement was computed by averaging the standard deviation of the relatedness scores for each pair [9]. The resulting value (inverse) agreement was 0.76—i.e., on average, the judgement of the annotators varied ±0.76 rating points around the final score assigned to each pair. Table 2 lists the distribution of the pair of sentences according to their relatedness scores.

**Table 2:** Statistics on the SICK corpus [9].

**Relatedness Scores**	**Number of pairs**
[1–2) range	925 (9%)
[2–3) range	1,380 (14%)
[3–4) range	3,904 (39%)
[4–5] range	3,718 (38%)
Total	9927

In order to investigate the applicability of such an approach to consolidating scientific artefacts in EBM, we performed a separate set of experiments using the NICTA-PIBOSO corpus [6]. The corpus comprises 10,000 sentences retrieved from publication abstracts representing studies that follow the Evidence Base Medicine (EBM) treatment practice. Each sentence is classified into one of the six types of scientific artefacts defined by the PIBOSO annotation scheme—i.e., Population, Intervention, Background, Outcome, Study Design and Other. Our ultimate goal is to consolidate sentences carrying the same rhetorical type, in order to build a knowledge network of key statements in the EBM domain. Such a network of scientific artefacts will alleviate the problem of finding similar studies and will help clinicians to effectively retrieve adequate evidence as a foundation for their decision-making. The focus of our study was on the 5 main PIBOSO types, i.e., omitting the ‘Other’ type, since its role is simply to mark any other sentences that do not fit into the main scheme. Table 3 lists the distribution of sentences in the NICTA-PIBOSO corpus according to their underlying types.

**Table 3:** The statistics of the NICTA-PIBOSO corpus.

**Sentence Type**	**Number of Sentences**	**Number of pairs of sentences**
Background	2,557	3,267,846
Intervention	690	237,705
Outcome	4,523	10,226,503
Population	812	329,266
Study Design	228	25,878

For each type of scientific artefact (e.g., Intervention, Outcome, etc) we created a corpus by pairing its instances in the NICTA-PIBOSO corpus. This results in five corpora, with their stats being listed in Table 3. The similarity value for each pair of sentences has been computed by applying the regression model trained on the SICK training corpus. In order to evaluate the scores, we randomly selected 50 pairs from each of the five corpora and asked five annotators (one clinical geneticist, two researchers and two PhD students) to assess their similarity using the same approach and annotation guidelines used to create the SICK corpus. For completeness purposes, since sentences in the NICTA-PIBOSO corpus may carry multiple rhetorical roles, we adapted the guidelines to include detailed examples on different types of pairs of scientific artefacts and the effective factors in identifying and assessing their similarity, e.g., the quantitative elements in Population pairs, the dimension of the study in Study Design pairs, etc. The final similarity scores for each pair was then calculated as the average of all the annotator scores.

### Regression models

Regression models have been trained using corresponding implementations from WEKA toolkit [27]. We report the experimental results using the Pearson correlation, in conjunction with the mean square error. The experimental setup included two phases: (i) a 10-fold cross-validation, aimed at understanding the added-value brought by different feature sets; and (ii) a testing phase on the blind test data.

We investigated the performance of a number of learning algorithms for predicting the relatedness scores for pairs of sentences. Here, we briefly describe the algorithms employed:

- **M5Rules** is a rule-based approach for regression problems that builds rules from regression trees. It iteratively produces a decision list using separate-and-conquer. Each iteration builds a model tree using M5 and transforms the “best leaf” into a rule [28].
- **Reduced-error Prune Tree (RepTree)** is a fast decision tree learner. It builds a decision/regression tree using information gain/variance and prunes it using Reduced-Error Pruning (with backfitting) [29].
- K* is an instance-based classifier—i.e., a test instance is assigned to a class based on the classes associated with similar training instances—where similarity is determined by a specified function. K* employs an entropy-based distance function [30].
- **Regression by discretisation** is a regression scheme that employs any classifier on a copy of the data that has the class attribute discretised. The predicted value is the expected value of the mean class for each discretised interval (based on the predicted probabilities for each interval).

Finally, for completeness purposes, below lists the different external resources we have used to compute in particular the semantic similarity features:

- Distance-based measure to estimate the relatedness of two terms have been retrieved from the Wordnet library described in [31].
- Structural similarity of sentences has been computed based on parse trees produced with the Stanford Parser [32].
- Word sense disambiguation approach employed WordNet∷SenseRelate∷AllWords [33] using its Perl implementation. The Lesk algorithm [24] has been employed in finding the best sense of each word of a sentence from the Wordnet dictionary.
- Distributional semantics (DS) based measures were computed using an index compiled from the Wikipedia’s English articles corpus. The actual DS feature values have been retrieved via the SemanticVector library [34], which was configured to produce term vectors based on Hyperspace Analogue to Language (HAL) vector space model [35]. There are a number of parameters in HAL model which can affect the resulting term vectors. In our experiment, by following an empirical analysis and learning from the literature [36, 37], we employed the default value of each of these parameters. For example, the size of the sliding context window, including the focus term as its center, was set to its default (i.e. 5). Also, the dimension of each term vector was set to its default of 200 (i.e., each word is represented by a vector with 200 dimensions).

## Experimental results

### Results on the SICK corpus

Table 4 shows the experimental results achieved by the selected regression approaches as well as the baseline approach. More concretely, it shows the results associated with the four algorithms introduced above, in addition to a set of results achieved by regression by discretisation / classification, and finally by an ensemble method. The baseline correlation is the value of a naive comparison of the overlapped words of pairs of sentences on the training data. Among the four regression algorithms the M5Rules was able to predict the closest similarity scores to the human annotated scores. M5Rules creates rules from a model tree by iteratively selecting the best leaves and transforming them into rules. The second best correlation was achieved by the RepTree model which is a reduced-error pruning based regression tree. It can be observed that the tree-based models are able to better recognise patterns from numerical similarity features.

**Table 4:** Evaluation of regression algorithms on 10-fold cross-validation on the SICK training corpus.

**Algorithm**	**Pearson Correlation**
Baseline Approach	
Baseline	0.63
Regression Algorithms	
M5 Rules (M = 10)	0.7705
RepTree (N = 2)	0.7483
K* (B = 30)	0.7391
Linear Regression	0.7055
Regression By Classification	
Regression by Random Forest (I = 150, #Bins = 10)	0.8139
Regression by KNN (K = 10, #Bins = 10)	0.7539
Regression by Naïve Bayes (#Bins = 10)	0.6529
Regression Ensemble	
Ensemble of Bagging (RepTree), Random SubSpace(K*), and Regression by Discretisation (Random Forest)	0.8268

In regression by classification, range-based continuous scores are discretised to nominal values, a classifier is used to categorise the instances based on these nominal values, followed by the application of a regression method to predict the final relatedness score. Different base classifiers and ranges can be considered in this approach. In our experiments, we discretised the continuous range of 1 to 5 scores into 10 bins. The best results have been achieved by applying Random Forest as the base classifier. It again shows the efficiency of the tree based models in aligning each numerical representation of similarity features to a proper overall similarity score for a pair of sentences. Furthermore, Random Forests are more robust with respect to noise, which is important in our setting since each of our similarity features carry small amounts of information about the whole similarity of a pair sentences.

Finally, the ensemble of regressors is composed of three meta-regressors: bagging, random SubSpace, and regression by discretisation. Regression by discretisation / classification follows the exact same methodology as above. The bagging strategy uses RepTree as its first level regressor (as RepTree algorithm performed better than M5Rules in the bagging strategy), while the random SubSpace approach employs K* algorithm. The final outputs of the ensemble are the average of the prediction values of the regressors. This ensemble gained the best correlation among all the models. Its evaluation over the training data via 10-fold cross validation achieved a correlation score of 0.8268, i.e., almost 0.20 points better than the baseline.

In order to gain a deeper understanding of the impact of diverse features on the final regression results, we performed a standard 10-fold cross validation through leave-one-out features as well as the evaluation of each feature set individually. Table 5 lists the results achieved by leaving out the feature set heading each row. The feature sets analysed here can be mapped onto the categories listed in Table 1. As learning method we used the model that achieved the best results in the full 10-fold cross validation—i.e., the ensemble of regressors.

**Table 5:** Analysis of effects of different similarity measures—Pearson Correlation results for 10-fold cross-validation using Leave-one-Out feature strategy (i.e. the model is trained on all features except the one mentioned in each row) and results for each measure individually (i.e. the model is trained only for the mentioned feature).

**Features**	**Leave one feature set out**	**Features individually**
Naive features	0.8266	0.7645
Window-based features	0.8266	0.7218
Other syntactic features	0.8183	0.7061
Sentence structure features	0.8272	0.5129
Basic semantic features	0.818	0.7543
Words Synonymy features	0.826	0.6538
Word-sense features	0.8237	0.7277
Semantic space models features	0.8256	0.7159

It can be observed that there are no considerable decreases in the performance when leaving any of the feature sets out. The feature set that has a higher impact (i.e. lower leave one feature set out result as well as higher individual positive impact) is the basic semantic similarity feature. In addition, the ensemble model predicts better similarity scores by only training over naive features set with 0.7645 correlation, while leaving this set out does not have considerable impact on the overall performance with only 0.0002 reduction in the model’s correlation.

Overall, it is worth noting that the structural features appear to have an overall negative impact, since the ensemble experiences a 0.0004 improvement in Pearson correlation (from 0.8268 to 0.8272). A cause for this phenomenon may be the over-simplification introduced by the reduced parse trees, which fail to encode more specific structural characteristics. The weakness of this feature set also can be observed in the performance of the model when it only trains on this set (with only 0.5129 correlation). To better examine the impact of the structural features, we removed this set from test set as well. Although leaving the structural features led to better correlation on 10-fold cross validation, they still have positive impact on the model when it is evaluated over the separate test data set (with 0.8198 correlation with absence of structural features from test set vs. 0.8207 correlation with their presence along with all the other features).

### Results on the NICTA-PIBOSO corpus

As mentioned in the previous section, we created five corpora using the NICTA-PIBOSO corpus, corresponding to the five rhetorical types captured by the PIBOSO scheme. Sentences in these corpora have been classified using the best regression method, with a subset of the scored sentences evaluated by five annotators. The average of the inter-annotator agreement is listed in Table 6. It can be observed that, except from Annotator 1, the other four annotators have more than 0.77 agreement. The worst agreement occurs between Annotator 1 and Annotator 2 with 0.62 correlation. The overall average correlation between annotators is 0.76 which shows good inter-annotator agreement. Furthermore, the details of class-based inter-annotator agreements are provided in the Supporting Information S1 Table document.

**Table 6:** Inter-annotator agreement.

	**Annotator agreement (pairwise Pearson correlation)**					
	**Annotator1**	**Annotator2**	**Annotator3**	**Annotator4**	**Annotator5**	**Average**
**Annotator1**	-	0.62	0.70	0.67	0.68	0.67
**Annotator2**	0.62	-	0.85	0.77	0.81	0.76
**Annotator3**	0.70	0.85	-	0.86	0.82	0.81
**Annotator4**	0.67	0.77	0.86	-	0.84	0.78
**Annotator5**	0.68	0.81	0.82	0.84	-	0.79
	**Overall Average**					0.76

In addition, to test the quality of the annotated scores, we measured the (inverse) inter-rater agreement by calculating the average of the standard deviation of similarity scores for each sentence pair. The average of the standard deviation for each corpus is shown in Table 7. It can be observed that the average values are relatively close to zero which shows strong agreement among annotators. The annotators gave the closest scores to each other on Intervention pairs, the average standard deviation is only 0.18, while their scores on Study Design differed the most with an average standard deviation of 0.44. Table 7 also shows the class based inter-annotator agreements in Pearson correlation. It can be observed that the least agreement among annotators occurs over Study Design pairs with only 0.53 average correlation. For Background and Outcome pairs there are strong consensuses with 0.87 and 0.84 correlations respectively. In addition, Population and Intervention pairs are also annotated with high agreements of 0.79 and 0.78 respectively.

**Table 7:** Evaluation of semantic similarity approach over EBM scientific artefacts.

	**Class-based sub-corpora of pairs**				
	**Background**	**Intervention**	**Outcome**	**Population**	**Study Design**
**Average standard deviation(of annotations scores)**	0.25	0.18	0.3	0.32	0.44
**Average inter-annotator agreements(Pearson correlation)**	0.87	0.78	0.84	0.79	0.53
**Pearson correlation of systems’ predictions and annotators’ scores**	0.90	0.57	0.84	0.12	0.56

To evaluate the predicted scores, which are produced by the trained model over the general English (SICK) corpus, we computed their Pearson correlation coefficient with the human annotated scores. As shown in Table 7, the predicted similarity scores for Background and Outcome pairs were closer to their human annotated scores with 0.90 and 0.84 correlations, respectively. In addition, the Intervention and Study Design scores have also achieved a relatively good correlation with scores of 0.57 and 0.56, respectively. On the other hand, the model’s predictions for Population pairs varied the most from their manually annotated scores. This behaviour of the model can be caused by the lack of domain-specific and class-based similarity measures, such as quantities for Population pairs, medical terms for Intervention pairs, etc. In the current approach, only generic features have been used to model the similarity of the sentences from general a English perspective. By comparing the outcome of the predictor model with the inter-annotator agreement, it can be observed that the model performs better when there are high agreement between the given scores by the annotators and when the sentences are closer to general English sentences mostly in terms of grammatical structure and vocabulary (e.g., for Background and Outcome pairs). As a remark, for Study Design class, where there is only 0.53 inter-annotator agreement, the model shows a robust behaviour by having 0.56 correlation between prediction and the given scores by the annotators.

Further experiments have been performed on domain specific pairs to investigate the contribution of each of the features on estimating the semantic similarity of the EBM sentences. Table 8 lists the performances of the model over each of the classes when it is trained on each of the mentioned similarity measures individually. It can be observed that the two sets of naive and other syntactic features have the most positive impact on the performance of the model for most classes. This shows the robustness of these features in formulating the similarity of sentences on both the general English domain and a specific domain such as EBM. In addition, by only training on naive features, the ensemble is able to perform better in predicting similarity scores for Intervention and Population pairs, comparing to when it is trained on all the features, from 0.57 to 0.62 and from 0.12 to 0.208, respectively. On the other hand, those features that rely on generic external resources, such as Wordnet for basic semantic, word synonymy, and word-sense features, and the Wikipeida articles as the base of indexing of terms in semantic space model features, have mostly negative impact on applying the generic regression model on the domain specific pairs. The reason for this deterioration is that these generic resources do not contain the special scientific terms and hence they do not provide any semantic information about them. As a result, those features that employ general syntactic specifications of the sentences have more positive impact when using a trained regression model over general English data on predicting similarity for a domain specific pairs.

**Table 8:** Analysis of effects of different similarity measures when the model is trained only on the mentioned features.

**Features**	**Background**	**Intervention**	**Outcome**	**Population**	**Study Design**
Naive features	0.8741	0.6231	0.7609	0.208	0.0435
Window-based features	0.5992	0.55.88	0.7954	0.1198	0.4042
Other syntactic features	0.8815	0.6992	0.721	-0.3219	0.398
Sentence structure features	-0.2688	-0.2521	0.0498	0.141	0.0652
Basic semantic features	0.8053	0.4235	0.6894	-0.0553	0.0555
Words Synonymy features	0.8038	0.5996	0.5908	0.0703	0.099
Word-sense features	0.6009	0.1709	0.6483	-0.1419	0.1976
Semantic space models features	0.7936	0.3122	0.7016	-0.0099	-0.1508

## Discussion

### Error analysis

**Fig 4:**
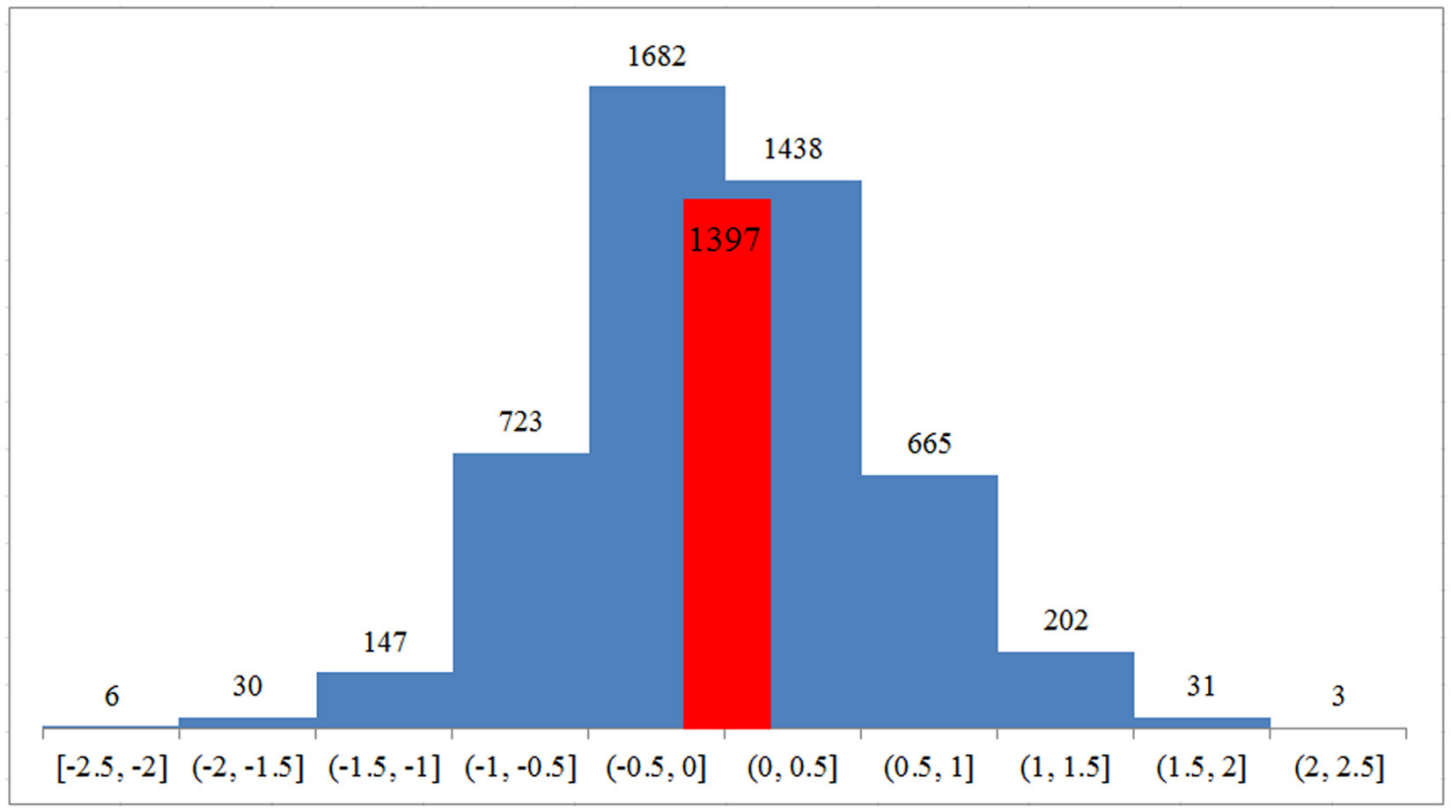
The error distribution of the ensemble predictions on SICK data.

Fig 4 and Table 9 depict the distribution of the prediction error of our ensemble on the SICK test dataset. The distribution reflects the number of errors performed by the ensemble in a set of intervals denoting the difference between the predicted and the actual value. It can be observed that 3,120 instances (i.e., 63.32% of the test dataset) have been predicted with a difference less than ±0.5 from the actual similarity score. Furthermore, 1,397 instances (i.e., 28.35% of the test dataset) have been predicted within ±0.2 of the actual similarity score and only 1.4% of the test instances have been predicted with more than ±1.5 from real score. One lesson learned was that the ensemble tends to assign scores lower that the real ones, with 2,588 instances (i.e., 52.53%) having a negative deviation from the real values and the rest of 2,339 (i.e., 47.47%) having a positive deviation.

**Table 9:** Prediction errors from the ensemble model.

**Error ranges**	**No. of instances within the error range**	**% of occurrences within the error ranges**
[-2.5, -2]	6	0.1%
(-2, -1.5]	30	0.6%
(-1.5, -1]	147	3%
(-1, -0.5]	723	14.7%
(-0.5, 0]	1682	34.1%
(0, 0.5]	1438	29.2%
(0.5, 1]	665	13.5%
(1, 1.5]	202	4.1%
(1.5, 2]	31	0.6%
(2, 2.5]	3	0.1%
Total	4927	100%

In order to gain a deeper understanding in the ensemble’s behaviour with respect to the classes of similarity, we split the test dataset into ranges of similarity—i.e., [1, 2), [2, 3), [3, 4), and [4, 5]. Note that the similarity scale ranged from 1 to 5, with 1 being the lowest similarity and 5 being the highest. Table 10 lists the associated results.

**Table 10:** Evaluation of the ensemble model on the test set, split onto the four score ranges.

**Score ranges**	**# instances**	**Pearson correlation**	**# underestimated predictions**	**# overestimated predictions**	**Lowest deviation**	**Highest deviation**
[1,2)	452	0.6646	16 (4%)	436 (96%)	-0.593	2.383
[2,3)	676	0.304	105 (16%)	571 (84%)	-1.69	2.086
[3,4)	1966	0.1828	843 (43%)	1123 (57%)	-1.85	1.849
[4,5]	1833	0.5649	1623 (89%)	210 (11%)	-2.455	0.564

It can be observed that the ensemble performs better for pairs of sentences that are either very similar (i.e., in the range [4,5]) or not at all similar (in the range [1,2))—i.e., the pairs found at the two ends of the similarity scale. The Pearson correlation values for these cases is 0.6646 and 0.5649 respectively—two to three times higher than the correlation associated with pairs in the grey area—i.e., [2,3) and [3,4). From a different perspective, the ensemble overestimates the similarity of the sentences that are less similar and underestimates the similarity for those that are more similar. For example, 96% of the predictions for the [1,2) range and 84% of predictions for [2,3) range are overestimated, with the highest deviation being 2.383 and 2.086 respectively. At the other end, 89% of the instances in the range [4,5] are underestimated, with the lowest deviation being -2.455. The pairs in the range [3,4) are almost equally split between overestimation and underestimation.

Below we present a series of both positive and negative prediction examples, with a focus on underestimate and overestimated sentence pairs, as well as those that have been very accurately predicted.

- Sentence pair: “*A cat is looking at a store counter*.” vs. “*A dog is looking around*.”; Actual score: 1.1; Predicted score: 3.34; Deviation (Prediction—Actual): 2.24
- Sentence pair: “*An airplane is taking off*.” vs. “*A plane is landing*.”; Actual score: 4.4; Predicted score: 2.27; Deviation: -2.13
- Sentence pair: “*A woman is cutting a vegetable*.” vs. “*A woman is slicing a vegetable*.”; Actual score: 4.7; Predicted score: 4.7 (4.696); Deviation: 0

The first example denotes a pair of highly overestimated sentences, with the actual score being 1.1 (i.e., highly dissimilar according to the human annotators) and the prediction deviation being 2.24 (i.e., found fairly similar by the ensemble—score: 3.34). The two sentences share the same predicate / action (“*is looking*”) and have different (yet, to some extent similar) pre-verb component (cat vs. dog) and post-verb component (store corner vs. around). Given the exact match in predicate, the very high structural similarity and the semantically similar pre-verb components (both cat and dog are animals) the ensemble scored the pair with an average similarity (3.34). In practice, this score is arguably correct, although it differs significantly from the human annotators perspective.

The second example is opposite to the previous one—i.e., two sentences deemed highly similar by human annotators (actual score of 4.4) and predicted as less similar by the ensemble (score 2.27 and, thus a deviation of -2.13). As above, both sentences have a high structural similarity and they share the head of the verb phrase. They are, however, different in the verb participle (although the actions are semantically similar) and in the actual lexical representation of the pre-verb component (although they both represent the same thing). Here, we can infer that both the synonymy features as well as the distributional semantics ones have failed to find correspondences between the pre-verb components and the participles, which led to the low similarity prediction score.

Finally, the last example shows a perfect match between the predicted and the actual scores. This is an example where the participle synonymy was successfully recognised.

### Related Work

To date, several approaches have been proposed to quantify the similarity of pairs of sentences—all on general English and none specifically on biomedical text. Below, we discuss some of the latest solution, with a focus on the set of features employed for similarity computation. The performance comparisons with the state-of-the-art will be presented in the following section.

Zhao et al. [10] proposed a supervised machine learning framework to compute relatedness between pairs of sentences, i.e., ECNU system. They use seven sets of similarity features including sentence length features, surface text similarity, semantic similarity, grammatical relationship, text difference measures, string features, and corpus-based features. These features have been aggregated as part of five different supervised learning algorithms, such as, Support Vector Machine (SVM), Random Forest, Gradient Boosting, k-nearest neighbours (kNN), and Gradient Decent. In addition, they have also tested a majority voting ensemble comprising these five algorithms. Finally, they attempted to utilise the unlabelled test data in the training process by employing a semi-supervised approach—i.e., the CoReg algorithm, which applies a co-training strategy with two kNN regressors.

The Meaning Factory is another system for determining semantic similarity of sentences [11]. It quantifies relatedness based on syntactic and semantic similarity measures. From a syntactic perspective, the system measured word overlap, discourse representation structure and sentence length. It also employed a range of semantic features, such as, Wordnet concept similarities, Compositional Distributional Semantics, and synsets overlap and distance. A first-order logic model has also been built using these features. The supervised learning approach developed by the system was a Random Forest Regressor.

Jimenez et al. [14] proposed the UNAL-NLP system for addressing the semantic relatedness task. By considering sentences as sets of words, they employed the soft cardinality of different relational sets operations. The sets of features presented by their approach consisted of string matching, explicit semantic analysis, part-of-speech tags analysis, syntactic roles dependencies, in addition to features considering linguistic phenomena such as antonymy, hypernymy, and negation. This system used reduced-error pruning tree (REPtree) as the regression model, boosted with 20 iterations of bagging.

The Illinos-LH system [12] is another supervised approach for predicting semantic relatedness of sentences. Its features are based on distributional and denotational similarities as well as alignment methods. Their complete list of features includes negation, word overlap, denotational constituent similarity, distributional constituent similarity, alignment, unaligned chunk matching, antonymy, synonymy and hypernymy. The choice of learning method was a log-linear regression model.

Saric et al. [13] measured similarity of sentences by applying knowledge-based, corpus-based and parse dependency based features in their TakeLab system. These measures quantified the similarity of sentences using: n-gram overlap, weighted word overlap, greedy lemma aligning overlap, vector space sentence similarity, syntactic roles similarity, syntactic dependencies overlap, numbers overlap, Named Entity features, and normalised differences. They also applied a supervised learning algorithm, more specifically the support vector regression (SVR) model.

All of above-mentions approaches, including ours, have used syntactic attributes in their similarity computation. Such attributes included: words overlap, string matching, lexical and role-based similarities, and other text-level features. The values of these features were, however, calculated differently from one approach to another. For example, in terms of matching sequences of strings between two sentences, we use window-based measures performed at the word level, rather than at the character level, as done by [14] and [10]. The synonymy features in our approach have been calculated by using both Wordnet and FrameNet. Lai et al. [12] used Wordnet to match the number of synonyms in a pair of sentences—similar to our approach. However, no system has employed FrameNet for word-based similarity. Finally, while all existing solutions use word-based similarity computed based on vector space models, none of them considered these measures for effective words and their associated terms.

A comprehensive comparative overview of the features used by all systems is presented in Table 11. Different approaches used various terminologies to name their features and they categorised using different perspectives. In addition, none of the approaches uses the exact same strategy for computing the same feature. Hence, we grouped their proposed similarity features based on their high-level aim and tried to align them to ours, in order to provide a fair as possible comparison.

**Table 11:** Comparative overview of the features used by existing systems.

**Similarity Measures**	**Similarity Features**	**Approaches**					
		**Zhao**	**Bjerva**	**Jimenez**	**Lai**	**Saric**	**Ours**
Naive	Bags of words overlap	✓	✓		✓		✓
	Bags of lemmatised/stemmed words overlap	✓					✓
	Set similarity of lemmatised effective words				✓		✓
	Jaccard similarity of set of words/lemmas	✓					✓
	Cosine similarity of vectors of lemmatisedeffective words						✓
	Character subsequence/n-gram overlap	✓		✓		✓	
	Weighted word overlap	✓				✓	
	Numbers overlap					✓	
	Discourse Representation Structure overlaps		✓				
	POS tag based words comparison			✓			
Window-based	Windows of words overlap						✓
	Size of the longest shared window of words						✓
	Windows of effective words overlap						✓
	Size of the longest shared window of effective words						✓
	Windows of POS tags overlapand longest overlapped windows						✓
Other	Ratio of shared skipped bigrams					✓	✓
	Pairwise sentence polarity	✓		✓	✓		✓
	Ratio of sentence lengths	✓	✓			✓	✓
	Logical Model		✓				
	Alignment of lemma of words				✓	✓	
	Dependencies features	✓		✓		✓	
	Named Entity features					✓	
Sentence Structure	Ratio of number of clauses						✓
	Reduced parse tree overlap						✓
Basic Similarity	Role-based word-by-word similarity			✓		✓	✓
	Semantic similarity of effective words	✓					✓
	Cosine similarity IC vectors						✓
	Role-based POS tags alignment						✓
	Wordnet concepts difference		✓				
Synonymy	WordNet-based synonym similarity				✓		✓
	FrameNet-based synonym similarity						✓
	Antonymy			✓	✓		
	Hypernymy			✓	✓		
Sense Disambiguation	Normalised set similarity of best senses		✓				✓
	Category level similarity of best senses						✓
	Norm. set sim. of the best senses skipped bigrams						✓
	Explicit Semantic Analysis			✓			
Vector Space Model	Similarity of sets of associated terms						✓
	Cosine Similarity of Matrices of Associated Terms Vectors	✓	✓		✓	✓	✓
	Weighted textual matrix factorization	✓					
	Distributional/Denotational Constituent Similarity				✓		

### Comparison against the state of the art

Table 12 shows the comparison of the experimental results achieved by our approach against four of the state of the art systems on the SICK dataset. Our system has performed on par with the two top ranked systems (i.e. ECNU and The Meaning Factory). More concretely, the Pearson correlations for these top two solutions were 0.8279 and 0.8268 respectively, while our approach achieved 0.8207. In addition, from MSE perspective, our approach ranked third, although the differences are again small, ranges from 0.3223 of the The Meaning Factory approach to 0.3338 of our system. It is worth noting, however, that on a 5-fold cross validation on the training set, our system showed more robustness with only ±0.009 deviation over the evaluation iterations comparing to ±0.058 of the ECNU approach. The other systems did not report the results on the 5-fold cross validation. Although the difference in scores is small, this may lead to the conclusion that our approach suffers from a small degree of overfitting, while ECNU seems to generalise slightly better.

**Table 12:** Experimental results achieved by our approach in comparison to the state of the art.

**Approach**	**Pearson Correlation**		**Mean squared error (MSE)**
	**Test data**	**5-fold cross validation (CV±STD)**	**Test data**
**Our Approach**	0.8207	**0.8226±0.009**	0.3338
**ECNU [10]**	**0.8279**	0.807±0.058	0.3250
**The Meaning Factory [11]**	0.8268	-	0.3223
**UNAL-NLP [14]**	0.8043	-	0.3593
**Illinois-HL [12]**	0.799	-	0.3691

## Conclusion

In this paper, we proposed a supervised approach for quantifying semantic similarity of full sentences. We described a series of measures that model the similarity of a pair of sentences from diverse perspectives, including syntactic, structural, and semantic. These were then used as features for an ensemble regression model. The evaluation of this model on the SICK corpus has showed competitive results when compared to the state of the art approaches—e.g., 0.8207 Pearson correlation factor on the SICK test data.

Our goal is, however, to consolidate scientific artefacts in the EBM domain—or more concretely to quantify the similarity of sentences carrying the same rhetorical role (e.g., Intervention, Outcome, etc). Since there is no available gold standard for this domain, we have crafted one by creating pairs of sentences from statements annotated in the NICTA-PIBOSO corpus. The regression model trained on the general English corpus has then been applied on the biomedical data and the results have been manually validated by five human annotators. The outcome is encouraging, with the predicted similarities achieving Pearson correlation scores of 0.90, 0.84, 0.57 and 0.56, respectively, on Background, Outcome, Intervention, and Study Design when the human scores are used as ground truth. Population is the type most heavily affected by the lack of domain specific features.

Future work will focus on adapting some of the proposed features to the biomedical domain, in order to leverage the conceptual semantics of the biomedical concepts to bootstrap the sentence-based similarity.

## Supporting Information

S1 Text Glossary. Definition and exemplification of a series of terms used throughout the manuscript.
(PDF)

S1 Table Inter-Annotator Agreements. Inter-annotator agreement values split onto the 5 PIBOS categories.
(PDF)
