# Supplementary material for: A Supervised Approach to Quantifying Sentence Similarity: With Application to Evidence Based Medicine
Source: PLoS One. 2015 Jun 3;10(6):e0129392. doi: 10.1371/journal.pone.0129392 (PMC4454558; doi:10.1371/journal.pone.0129392)
Supplement: S1 Text — Definition and exemplification of a series of terms used throughout the manuscript. (PDF) [file pone.0129392.s001.pdf]

S1 Glossary

Below we provide the definition of and exemplify a series of technical terms used to describe our classification features. Examples are introduced based on the *Outcome A* sentence from the Introduction – i.e. "*No clinically relevant adverse events, such as urinary retention, respiratory disturbances, or wound infections were reported in the M-ADL group.*"

- **Bag of words of a sentence**: All constituent words of a sentence including multiple occurrences of the same word. For our example sentence the bag of words is: {no, clinically, relevant, adverse, events, such, as, urinary, retention, respiratory, disturbances, or, wound, infections, were, reported, in, the, M-ADL, group}.
- **Set of words of a sentence**: This is similar to the bag of words but it prevents duplications (i.e. multiple occurrences of the same word). The set of words associated with our example is: {no, clinically, relevant, adverse, events, such, as, urinary, retention, respiratory, disturbances, or, wound, infections, were, reported, in, the, M-ADL, group}.
- **Stemmed and lemmatised forms of words**: Both refer to the base form of a word but produced using a different process. Stemming aims to reduce the derived form of a word to its root version based on a fixed set of rules applied on the word's suffix without any background knowledge. Lemmatisation, on the other hand, has the same goal but with the use of additional information – in particular the part of speech of the word under scrutiny. In most cases both the lemma and the stem of a word are the same – e.g., the stem / lemma form of "disturbances" is "disturbance". In some cases, they are however different – e.g., the lemma of "training" is "training", while the stem is "train". As remark – our pipeline uses the Stanford Parser/CoreNLP toolkit to obtain lemmas and stems.
- **Effective words of a sentence**: The words that play a major role in a sentence. We consider words belonging to the nouns (NNs) and verbs (VPs) families as effective words. Words belonging to the other part-of-speech groups (such as determiners) are not included. For the above sentence example, the set of lemmatised effective words is: {event, retention, disturbance, wound, infection, be, report, group}.
- **Parse tree and Typed Dependencies**: A parse tree represents the syntactic structure of a sentence in the form of a rooted tree. Typed dependencies represent grammatical relations between words in a sentence. The parse tree and the typed dependencies of the example sentence are shown in Figure S1 below.
- **Skipped bigrams***:* All possible pairs of words of a sentence regardless of the position of the words. In our example, the skipped bigrams of the term "no" are: no-clinically, no-relevant, no-adverse, no-events, no-such, no-as, no-urinary, no-retention, no-respiratory, etc.
- **Information Content (IC)**: is a probabilistic measure that denotes the frequency of the occurrence of a concept/term in a corpus. A unique concept/term would be assigned higher IC value while a less specific concept has a low value.

**Typed Dependencies:**	**Parse Tree**
neg(events-5, No-1)<br>advmod(relevant-3, clinically-2)<br>amod(events-5, relevant-3)<br>amod(events-5, adverse-4)<br>nsubjpass(reported-19, events-5)<br>mwe(as-8, such-7)<br>prep(events-5, as-8)<br>amod(retention-10, urinary-9)<br>pobj(as-8, retention-10)<br>amod(disturbances-13,respiratory-12)<br>appos(retention-10, disturbances-13)<br>cc(retention-10, or-15)<br>amod(infections-17, wound-16)<br>conj(retention-10, infections-17)<br>auxpass(reported-19, were-18)<br>root(ROOT-0, reported-19)<br>prep(reported-19, in-20)<br>det(group-23, the-21)<br>amod(group-23, M-ADL-22)<br>pobj(in-20, group-23)	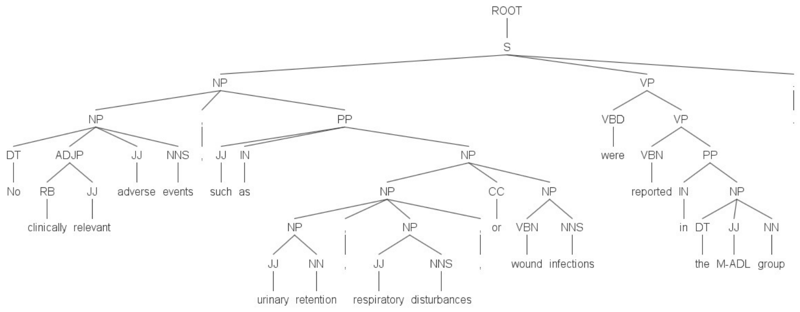

Fig S1. Typed dependencies and parse tree for a sample sentence

- **Associated Terms/Vectors:** Terms that are contextually co-occurring within a given corpus are named associated terms. These associated terms are usually obtained based on a vector space model. For example, the word "event" has as associated terms (based on the Wikipedia Corpus): game, competition, scene, movie, program, tournament, etc. The associated term vectors are the numerical representations of words based on the same vector space model. For instance the word "event" is represented with the following term vector: [0.016995998; -0.075350426; 0.010145959; -0.004498533; -0.0057947207; 0.0030134628; -0.0064116227; 0.01705665; 0.017748065; 0.038618762 …].
- **Synset**: a set of synonyms from WordNet (a lexical resource of English words and their semantic relationships). For example, one of the synsets of the word "event" is: SYNSET{SID-11410625-N : Words [W-11410625-N-1-consequence, W-11410625-N-2-effect, W-11410625-N-3-outcome, W-11410625-N-4-result, W-11410625-N-5-event, W-11410625-N-6-issue, W-11410625-N-7-upshot]}.
- **FrameNet**: a lexical database of English words and their meaning in the form of semantic frames.
