# Supplementary material for: A Supervised Approach to Quantifying Sentence Similarity: With Application to Evidence Based Medicine
Source: PLoS One. 2015 Jun 3;10(6):e0129392. doi: 10.1371/journal.pone.0129392 (PMC4454558; doi:10.1371/journal.pone.0129392)
Supplement: S1 Table — Inter-annotator agreement values split onto the 5 PIBOS categories. (PDF) [file pone.0129392.s002.pdf]

S2 Inter-Annotator Agreements

In this section the inter-annotator agreement per each of the five PIBOS classes is provided. Table 1 to Table 5 show these agreements based on pairwise Pearson correlation between annotators.

Table 1. Inter-annotator agreement for Intervention pairs

	Annotator 1	Annotator 2	Annotator 3	Annotator 4	Annotator 5	Average
**Annotator 1**	-	0.50	0.60	0.57	0.83	**0.62**
**Annotator 2**	0.50	-	0.89	0.95	0.79	**0.78**
**Annotator 3**	0.60	0.89	-	0.97	0.86	**0.83**
**Annotator 4**	0.57	0.95	0.97	-	0.83	**0.83**
**Annotator 5**	0.83	0.79	0.86	0.83	-	**0.83**
Overall average						**0.78**

Table 2. Inter-annotator agreement for Population pairs

	Annotator 1	Annotator 2	Annotator 3	Annotator 4	Annotator 5	Average
**Annotator 1**	-	0.59	0.71	0.71	0.74	**0.69**
**Annotator 2**	0.59	-	0.80	0.83	0.89	**0.78**
**Annotator 3**	0.71	0.80	-	0.89	0.88	**0.82**
**Annotator 4**	0.71	0.83	0.89	-	0.86	**0.82**
**Annotator 5**	0.74	0.89	0.88	0.86	-	**0.84**
Overall average						**0.79**

Table 3. Inter-annotator agreement for Study Design pairs

	Annotator 1	Annotator 2	Annotator 3	Annotator 4	Annotator 5	Average
**Annotator 1**	-	0.44	0.64	0.38	0.14	**0.40**
**Annotator 2**	0.44	-	0.76	0.39	0.50	**0.52**
**Annotator 3**	0.64	0.76	-	0.64	0.57	**0.65**
**Annotator 4**	0.38	0.39	0.64	-	0.81	**0.55**
**Annotator 5**	0.14	0.50	0.57	0.81	-	**0.50**
Overall average						**0.53**

Table 4. Inter-annotator agreement for Background pairs

	Annotator 1	Annotator 2	Annotator 3	Annotator 4	Annotator 5	Average
**Annotator 1**	-	0.83	0.86	0.89	0.87	**0.86**
**Annotator 2**	0.83	-	0.89	0.82	0.97	**0.88**
**Annotator 3**	0.86	0.89	-	0.90	0.86	**0.88**
**Annotator 4**	0.89	0.82	0.90	-	0.82	**0.86**
**Annotator 5**	0.87	0.97	0.86	0.82	-	**0.88**
Overall average						**0.87**

Table 5. Inter-annotator agreement for Outcome pairs

	**Annotator 1**	**Annotator 2**	**Annotator 3**	**Annotator 4**	**Annotator 5**	**Average**
**Annotator 1**	-	0.74	0.70	0.80	0.84	**0.77**
**Annotator 2**	0.74	-	0.90	0.84	0.89	**0.84**
**Annotator 3**	0.70	0.90	-	0.89	0.93	**0.86**
**Annotator 4**	0.80	0.84	0.89	-	0.87	**0.85**
**Annotator 5**	0.84	0.89	0.93	0.87	-	**0.88**
Overall average						**0.84**
